# CD90 is regulated by notch1 and hallmarks a more aggressive intrahepatic cholangiocarcinoma phenotype

**DOI:** 10.1186/s13046-022-02283-8

**Published:** 2022-02-16

**Authors:** Serena Mancarella, Grazia Serino, Isabella Gigante, Antonio Cigliano, Silvia Ribback, Paola Sanese, Valentina Grossi, Cristiano Simone, Raffaele Armentano, Matthias Evert, Diego F. Calvisi, Gianluigi Giannelli

**Affiliations:** 1 National Institute of Gastroenterology “S. de Bellis”, Research Hospital, Via Turi 27, 70013 Castellana Grotte, Italy; 2 Institute of Pathology, University of Regensburg, 93053 Regensburg, Germany; 3 Institute of Pathology, University of Greifswald, 17489 Greifswald, Germany

**Keywords:** Intrahepatic Cholangiocarcinoma, THY1/CD90, NOTCH pathway inhibition, Xenograft models

## Abstract

**Background:**

Intrahepatic Cholangiocarcinoma (iCCA) is characterized by a strong stromal reaction playing a role in tumor progression. Thymus cell antigen 1 (*THY1*), also called Cluster of Differentiation 90 (CD90), is a key regulator of cell–cell and cell–matrix interaction. In iCCA, CD90 has been reported to be associated with a poor prognosis. In an iCCA PDX model, we recently found that CD90 was downregulated in mice treated with the Notch γ-secretase inhibitor Crenigacestat. The study aims to investigate the role of CD90 in relation to the NOTCH pathway.

**Methods:**

THY1/CD90 gene and protein expression was evaluated in human iCCA tissues and xenograft models by qRT-PCR, immunohistochemistry, and immunofluorescence. Notch1 inhibition was achieved by siRNA. THY1/CD90 functions were investigated in xenograft models built with HuCCT1 and KKU-M213 cell lines, engineered to overexpress or knockdown *THY1*, respectively.

**Results:**

CD90 co-localized with EPCAM, showing its epithelial origin. In vitro, *NOTCH1* silencing triggered *HES1* and *THY1* down-regulation. *RBPJ*, a critical transcriptional regulator of NOTCH signaling, exhibited putative binding sites on the *THY1* promoter and bound to the latter, implying CD90 as a downstream NOTCH pathway effector. In vivo, Crenigacestat suppressed iCCA growth and reduced CD90 expression in the PDX model. In the xenograft model, Crenigacestat inhibited tumor growth of HuCCT1 cells transfected to overexpress CD90 and KKU-M213 cells constitutively expressing high levels of CD90, while not affecting the growth of HuCCT1 control cells and KKU-M213 depleted of CD90. In an iCCA cohort, patients with higher expression levels of *NOTCH1/HES1/THY1* displayed a significantly shorter survival.

**Conclusions:**

iCCA patients with higher NOTCH1/HES1/THY1 expression have the worst prognosis, but they are more likely to benefit from Notch signaling inhibition. These findings represent the scientific rationale for testing NOTCH1 inhibitors in clinical trials, taking the first step toward precision medicine for iCCA.

**Supplementary Information:**

The online version contains supplementary material available at 10.1186/s13046-022-02283-8.

## Background

Intrahepatic cholangiocarcinoma (iCCA) is a highly aggressive tumor developing in the biliary tract and the second most common primary hepatic cancer after hepatocellular carcinoma (HCC) [1]. In recent years, the global trend for iCCA has risen, reaching 2–3/100,000 inhabitants in most countries [2]. Survival rates for iCCA patients at 1, 3, 5-years are only 52.1, 21.7, and 11.2%, respectively [3]. The only curative treatment options for iCCA are partial hepatectomy or chemotherapy and radiotherapy. However, these therapeutic approaches are suitable only for patients with early-stage disease; in addition, the tumor frequently recurs. Treatments for advanced iCCA are limited, and the prognosis is poor [4]. The lack of more effective therapies is mainly due to the poor knowledge of the carcinogenesis mechanisms underlying iCCA development and progression and the lack of reliable biomarkers, predictive tools, and proper molecular classification. It has been proposed that the stroma reaction, particularly the interaction between iCCA cells and extracellular matrix components (ECM) of the surrounding microenvironment, plays a crucial role in disease progression (Coulouarn, Hepatology 2013). In this context, Thymus cell antigen 1 (*THY1*), also called Cluster of Differentiation 90 (CD90), is a 25–37 KD glycophosphatidylinositol (GPI)-anchored protein expressed in numerous cell types, including T cells, neurons, endothelial cells, and cancer-associated fibroblasts (CAFs). THY1 is a critical regulator of cell adhesion and communication, cell-ECM interaction and the immune and nervous systems [5, 6].

The latest findings report that in several tumors, including ovarian cancer, *THY1* is overexpressed in cancer stem cells (CSC) and the tumor microenvironment, enhancing proliferation and metastasis abilities [7]. Furthermore, in HCC, *THY1* expression is related to poor tumor differentiation, invasive properties, and dismal prognosis [8, 9]. In iCCA, CD90 was reported, in a single study by immunohistochemistry, to be associated with lymph nodes metastasis [10]. Nevertheless, no data explaining the role of CD90 at the molecular level in iCCA are yet available. Recently, performing a comprehensive transcriptomic analysis in a PDX model of iCCA, we discovered the downregulation of CD90 in mice treated with Crenigacestat [11]. This molecule is a selective NOTCH1 γ-secretase inhibitor (GSI), already tested in phase 1 clinical trial in patients with advanced or metastatic solid tumors including CCA (NCT02784795), and currently used in further clinical studies (https://clinicaltrials.gov/ct2/show/NCT02784795). NOTCH signaling plays an essential role in biliary development and regeneration, tubulogenesis, and survival of stem cell niches [12]. Aberrant and/or overexpression of NOTCH1 via *HES1* has been related to the development of iCCA in a mouse model [13]. The NOTCH cascade is initiated by the proteolytic cleavage of the γ-secretase enzyme that releases the NOTCH Intracellular Domain (NICD), which engages ligands of the Jagged or Delta-like families. NICD binds to the Recombination Signal Binding Protein For Immunoglobulin Kappa J Region (RPBJ) transcription factor in the nucleus, thus regulating its activity [14]. Inhibition of RBPJ activity led to the total inhibition of the NOTCH signaling pathway and the consequent reduction of iCCA development in the AKT/JAG-1 mouse model [15].

This study aims to investigate the function of CD90 in relation to the activated NOTCH signaling cascade and the potential clinical implications.

## Methods

### Cell lines and treatment

The HuCCT1, RBE, KKU-M213, and KKU-M156 human iCCA cell lines were purchased from ATCC or RIKEN. Regular monitoring with the MycoFluor™ Mycoplasma Detection Kit (ThermoFisher Scientific, Waltham, MA, USA) was performed to obtain a mycoplasma-free cell culture environment. The first two cell lines were cultured in DMEM (Dulbecco's Modified Eagle Medium), while the last two were cultured in RPMI, both supplemented with Sodium Pyruvate, Antibiotic–Antimycotic, Hepes, and 10% fetal bovine serum (FBS) (Thermo Fisher Scientific, Waltham, MA, USA). Crenigacestat (LY3039478, Selleckchem Chemicals, Houston, TX, USA) was used in in vitro and in vivo studies as GSI. Stock solutions were prepared in dimethylsulfoxide (DMSO) (Thermo Fisher Scientific, Waltham, MA, USA), and aliquots were stored at − 20 °C.

### Transient transfection of THY1 small interferingRNA (siRNA)

Mycoplasma-free KKU-M213, RBE, and KKU-M156 iCCA cell lines, after validation (Genetica DNA Laboratories, Burlington, NC, USA), were used for this study. Cell lines were maintained as monolayer cultures in Dulbecco's modified Eagle medium supplemented with 10% fetal bovine serum (FBS; Gibco, Grand Island, NY, USA), 100 U/mL penicillin, and 100 g/mL streptomycin (Gibco, Grand Island, NY, USA). Cells were grown for 12 h in complete medium, then serum-deprived for 24 h and treated with siRNA against NOTCH1 (# s453558; ThermoFisher Scientific Waltham, MA, USA), following the manufacturer's recommendations. Effects at 48 h after siRNA transfection are shown.

### Stable THY1 silencing and overexpression

For loss of function studies, the KKU-M213 cell line was transduced with Human shRNA lentiviral particles carrying 4 *THY1* specific sequences (A to D) and scramble control-shRNA sequence (OriGene Technologies, Inc., Rockville, MD 20,850, USA). Scramble control-shRNA sequence and *THY1*-shRNA sequence B were used in all the experiments involving *THY1* downregulation at a multiplicity of infection (MOI) 100.

For gain of function studies, the HuCCT1 cell line was transduced with human THY1-CMV-GFP and Lenti-CMV-GFP-2A-Puro-Blank Lentivirus (Aurogene, Italy) at MOI 40.

Both genetically modified cell lines were selected with puromycin dihydrochloride (Thermo Fisher Scientific, Waltham, MA, USA) at 1 µg/ml and 0.625 µg/ml for KKU-M213 and HuCCT1, respectively, to obtain stable *THY1* silencing and *THY1* overexpression, according to the manufacturer's instructions.

### Immunohistochemistry and immunofluorescence

For immunohistochemistry (IHC), formalin-fixed, paraffin-embedded (FFPE) tissues from 174 patients with iCCA, routinely collected for diagnosis purposes, were studied. Tumor sections of 4 μm were freshly cut and dried at 60 °C for 30 min. IHC analysis was carried out in sections after deparaffinization for 30 min and then rehydration in grades of alcohol. Antigen retrieval was performed at 90 °C for 20 min with Tris–borate-EDTA Buffer. An automated stainer (cat. K5007, Dako, Glostrup, Denmark) was used to stain the iCCA sections with the primary anti-CD90 antibody diluted 1:150 (Abcam, Cambridge, MA, USA). The Real Envision DAB Substrate Kit (DAKO) was used according to the manufacturer's instructions. CD90 expression was scored for all staining patterns, according to the staining intensity and the percentage of positively stained cells, by two independent, blinded pathologists.

Additionally, two groups (*n* = 10) of KKU-M213 scramble shRNA tumors treated with vehicle or Crenigacestat and two groups (*n* = 10) of HuCCT1 *THY1* +  + tumors treated with vehicle or Crenigacestat were stained by immunohistochemistry with AKT and pAKT (1:100 Cell Signaling Technologies, Massachusetts, USA).

For immunofluorescence (IF) staining, frozen tissues sections were fixed in a 1:1 acetone:chloroform solution, blocked with 2% bovine serum albumin solution, and incubated with anti-CD90, anti-αSMA, and anti-EPCAM antibodies (1:500, Cell Signaling Technologies, Massachusetts, USA). After washing, slides were incubated with secondary goat anti-rabbit immunoglobulin G H&L (Alexa Fluor 488, Thermo Fisher Scientific, Waltham, MA, USA).

For the fluorescence imaging of CD90, the cells fixed with PFA4% were permeabilized with 0.1% Triton X-100 in PBS in 2% bovine serum albumin for 30 min. After washing three times in PBS, the chamber slide were incubated with anti-CD90 antibody (1:500, Cell Signaling Technologies, Massachusetts, USA) in blocking solution for 1 h at 37 °C in a humid chamber. After washing three times in PBS, the chamber slide was incubated with secondary goat anti-rabbit immunoglobulin G H&L (1:50 Alexa Fluor 488, Thermo Fisher Scientific, Waltham, MA, USA) for 1 h at 37 °C in a humid chamber in the dark, then washed with PBS three times and covered with 4′,6-diamidino-2-phenylindole (DAPI)-supplemented antifade mounting medium VECTASHIELD (Vector Lab, Burlingame, CA, USA) to stain the nucleus. As a negative control, a well of the chamber slide was stained while omitting the primary antibody.

For the visualization of immunohistochemistry and immunofluorescence, images the Eclipse Ti2 microscope (Nikon Inc., Melville, NY) were used. For each sample, 5 images were captured in different positions, and staining was quantified using Image J analysis software.

### Spheroid formation assay

96-wells of the tissue culture plate were pre-coated with Matrigel Matrix, growth factor reduced, phenol-red free (Corning, Bedford, MA, USA). Refrigerated Matrigel is dispensed onto the cold surface of the wells and then kept at 37 °C to allow gelation. 2 × 10^3^ cells/well were resuspended separately in a refrigerated matrigel matrix to enable the embedding of the cells within the matrix upon gelation. After leaving the suspension overnight, we treated the cells with increasing concentrations of Crenigacestat (1–5-10 µM). The culture medium, with the treatment, was changed every two days. After 13 days, the spheroid cultures were stopped for a proliferation assay, CellTiter 96® AQueous One Solution Cell Proliferation Assay (Promega Italia s.r.l., Milan, Italy), a colorimetric method for determing cell viability.

### In vivo studies

In the present study, we examined the modulation of the CD90/*THY1* in our iCCA patient-derived xenograft (PDX) model treated with Crenigacestat, established in our previous work [11].

Two million stable CD90-silenced KKU-M213 cell lines or CD90 overexpressed HuCCT1 cell lines, or corresponding cells that received the empty vector, were subcutaneously injected into the flanks of 4–5-week-old females CD1 nude mice. Each mouse was monitored daily for clinical signs and mortality, and body weight was recorded twice a week. Tumor growth was assessed with a caliper once a week, evaluating tumor volume with the formula (mm^3^) = [length (mm) × width (mm)^2^]/2, where width and length are the shortest and the longest diameters. When the tumor masses volume reached approximately 70–100 mm^3^, the mice were randomly divided into 8 experimental groups of ten animals and administered Crenigacestat (8 mg/kg) or vehicle by oral gavage every 2 days for 35 days. At the end of the study, mice were sacrificed by cervical dislocation, and tumor masses were collected and frozen to -80 °C.

### RNA extraction

Total RNA was extracted using TRIzol® (Thermo Fisher Scientific, Waltham, MA, USA) in combination with the TissueLyser homogenizer (Qiagen, Hilden, Germany), according to the manufacturer's instructions. The RNA concentration was determined with the NanoDrop Spectrophotometer (Thermo Fisher Scientific, Waltham, MA, USA).

### Quantitative reverse-transcription real-time PCR (qRT-PCR)

cDNA was obtained starting from 1 µg of total RNA, using the iScript Reverse Transcription Supermix (Bio-Rad Laboratories, Hercules, CA, USA) according to the manufacturer's instructions. Quantitative PCR reactions were performed using SsoAdvanced SYBR green (Biorad Laboratories, Hercules, CA, USA) and the primers *THY1* Human PrimePCR™ SYBR® Green Assay ID: qHsaCED0036661 (Biorad Laboratories, Hercules, CA, USA) and Hs_*GAPDH*_1_SG QuantiTect Primer Assay ID: QT00079247 (Qiagen, Hilden, Germany). Gene Expression Assays for human NOTCH1 (Hs01062014_m1), HES1 (Hs00172878_m1), *THY1* (Hs06633377_s1), and β-actin (4333762 T) were purchased from Applied Biosystems (Foster City, CA, USA). Quantitative values for each gene were calculated by using the PE Biosystems Analysis software and expressed as number target (NT). NT = 2 − ΔCt, wherein the ΔCt value of each sample was calculated by subtracting the average Ct value of the target gene from the average Ct value of the β-Actin gene. Experiments were repeated three times in triplicate. For the screening of *AKT* Signaling, PrimePCR arrays (Cat #10,025,059, BioRad Laboratories, Hercules, CA, USA) was used. Real-time PCR was performed on the CFX96 System (Biorad Laboratories, Hercules, CA, USA). Comparative real-time PCR was performed in triplicate, including no-template controls. Relative expression was calculated using the 2^−ΔΔCt^ method. The ΔCt value of each sample was calculated by subtracting the average Ct value of the target gene from the average Ct value of the *GAPDH* gene.

### Chromatin immunoprecipitation

Chromatin isolated from KKU-M213, KKU-M156, and RBE cells was subjected to immunoprecipitation using the MAGnify Chromatin Immunoprecipitation System (492,024, Thermo Fisher Scientific, Waltham, MA, USA) according to the manufacturer's instructions. Chromatin was sonicated to a fragment length of about 500 bp and immunoprecipitated with 1 μg of RBPJ (Cell Signaling Technologies, Massachusetts, USA; Active Motif, Belgium, Germany), H3K4me3 (Abcam, Cambridge, MA), H3K27me3 (Abcam, Cambridge, MA, USA), and IgG (Cell Signaling Technologies, Massachusetts, USA) antibodies. The set of primers used for ChIP allows the amplification of target regions, including *RBPJ* consensus motifs (1 and 2) sites at—1000 bp (genomic position 119,424,535 – 119,424,508) from TSS at the 5' UTR region of the *THY* gene locus. Primer sequences can be provided upon request.

### Statistical and bioinformatic analysis

All experimental results are expressed as mean ± standard error of the mean (SEM). GraphPad Prism version 5.03 (GraphPad Software, San Diego, CA, USA) was used to calculate Unpaired Student's t-test and one-way analysis of variance (ANOVA). Differences were considered statistically significant at p values * *P* < 0.05, ** *P* < 0.01, and *** *P* < 0.001.

Each biomarkers distribution was compared using a graphical plot that shows the scatter distribution of the quantities across medians and interquartile ranges. Due to the non-normal distribution of the standard deviations of biomarker distribution, the Wilcoxon test on the median was used to compare the expression of the biomarkers in the tumor tissues vs. surrounding areas. Pearson correlation analysis was applied to reveal a linear association between each biomarker in the following combinations: *CD90* vs. *NOTCH1*, *CD90* vs. *HES1*, *HES1* vs. *NOTCH1*. To assess the statistical significance of monotonic associations between the variables, a *P* < 0.05 was used as a cut-off to reject the null hypothesis.

The whole patients' population was subdivided into two groups according to the median values of each biomarker, assayed both separately and summed. Subsequently, the non-parametric Kaplan–Meier method was used to explore the survival probability in months, comparing the two groups of patients (below median vs. above median) for each biomarker and all the biomarkers together. Log-rank test was applied to evaluate the equality of survival among categories, setting a statistical significance threshold of < 0.05.

*RPBJ* putative transcription factor binding motifs on the *THY1* gene promoter were predicted using the Eukaryotic Promoter Database New (EPDnew [16]; https://epd.epfl.ch).

The Ingenuity pathway analysis (IPA) software (Qiagen, Hilden, Germany) was used to uncover the canonical pathways and molecular networks in which modulated genes are involved.

## Results

We have previously reported that in a PDX model of iCCA, Crenigacestat treatment reduces tumor growth. However, in some in vitro experimental models, Crenigacestat did not affect either proliferation, survival, migration, and adhesion, either in 2D or 3D experimental conditions [11]. This apparent inconsistency prompted us to make a deeper investigation on the cancer stemness involved in cancer responsiveness/resistance.

### THY1/CD90 is expressed by epithelial cells in iCCA tissues

**Fig. 1:**
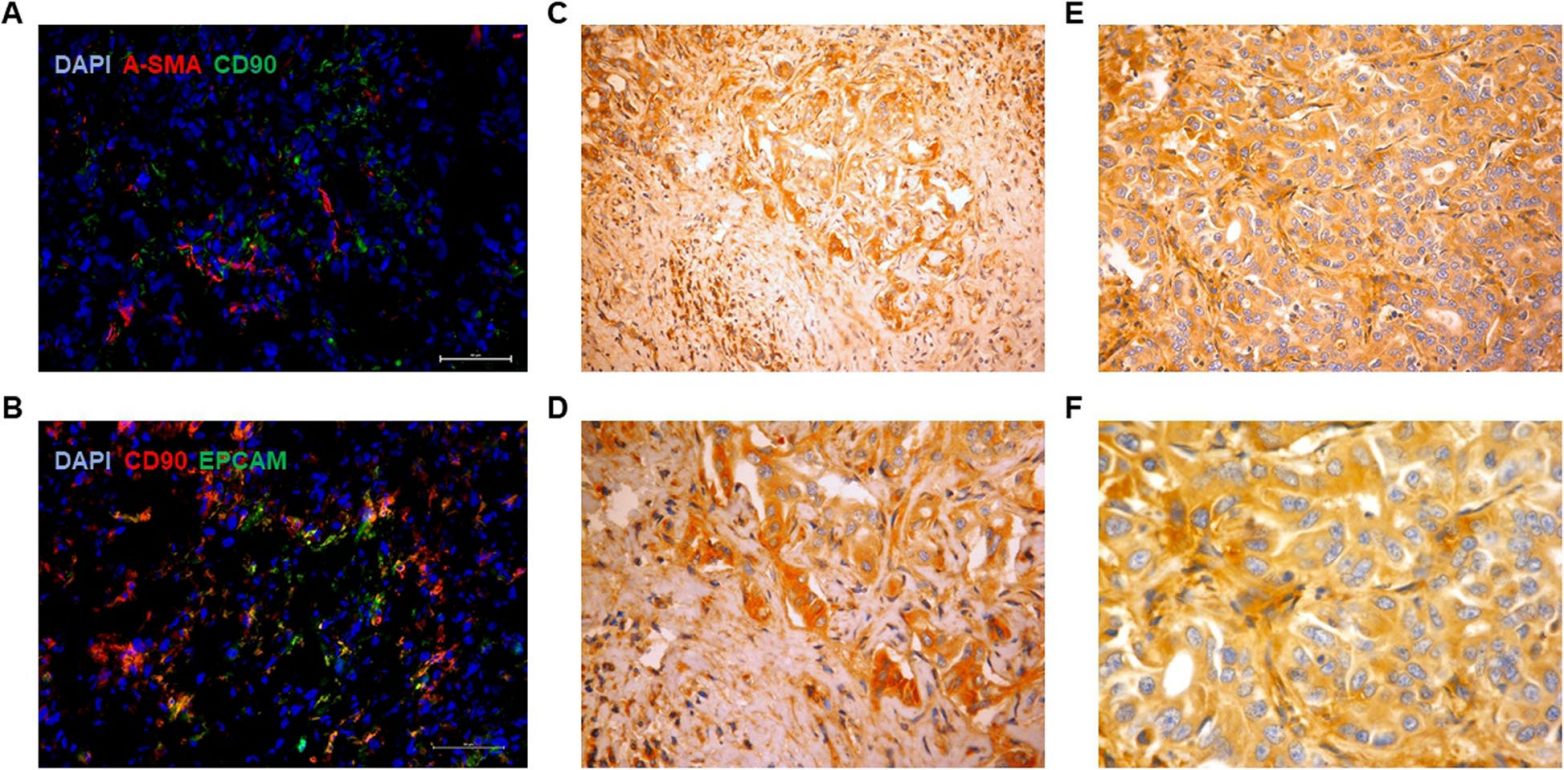
CD90 staining in PDX and human tissues. **A** CD90 does not co-localize with α-SMA (red), **B** but co-localizes with EpCam (green) on frozen PDX tissues, as shown by immunofluorescence staining. CD90 immunohistochemistry on human iCCA paraffin-embedded tissues at low (× 4) and high (× 20) magnification. Intense cytoplasmic positivity was detected in approximately 60% of the cells, independently of moderate **C-D** or poor **E–F** tumor differentiation

In all frozen iCCA PDX tissues sections, CD90 was co-localized with EpCAM, a specific molecular marker of epithelial cells (Fig. 1B) but not with alpha-smooth muscle actin (α-SMA), a marker of stromal cells such as myofibroblasts (Fig. 1A). The distribution pattern of CD90 was intracellular and on the plasma membrane, as observed in the microscopic fields. Similarly, in a collection of 174 human iCCA paraffin-embedded tissues, CD90 was predominantly expressed in the cytoplasm of the tumor epithelial cells, independently of moderate (Fig. 1C-D) or severe differentiation (Fig. 1E-F). These findings suggest that in the PDX model and patients' tissues, CD90 is present on epithelial but not on stromal cells, showing a similar intracellular and plasma membrane distribution.

### THY1/CD90 is a NOTCH target

**Fig. 2:**
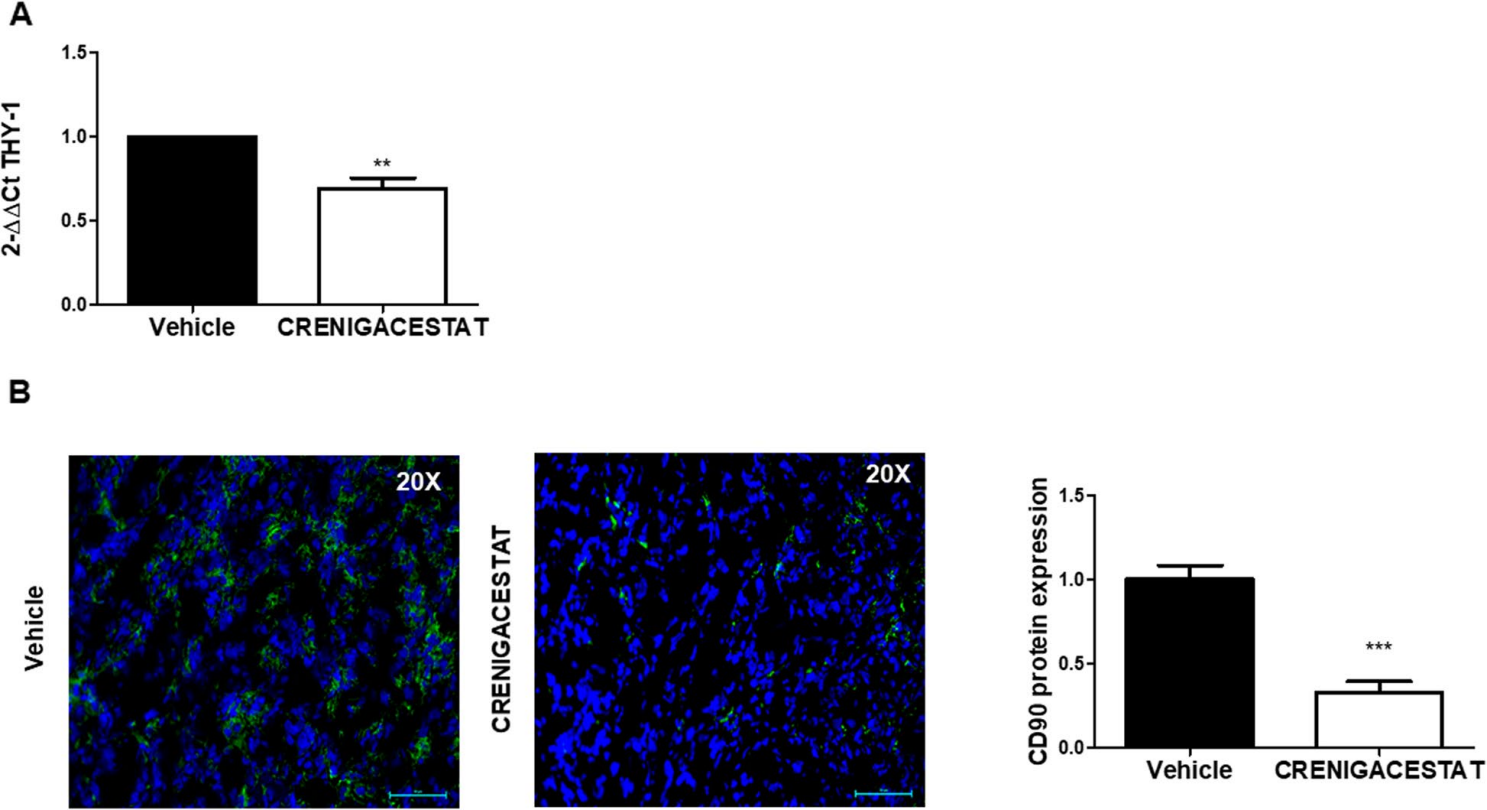
Crenigacestat decreases CD90 expression in iCCA PDX tissues. **A** THY1 is downregulated in iCCA PDX tissues following treatment with Crenigacestat. Fold-change -2.49 and FDR = 0.01. **B** Representative images with immunofluorescence staining show downregulation of CD90 expression. Staining quantification was calculated as the mean of three images per tissue from PDX mice treated with Crenigacestat compared to vehicle. Mean ± SEM (number of PDX mice treated with vehicle = 10, number of PDX mice treated with Crenigacestat = 10). **p < 0.01; ***p < 0.001 calculated with Mann Whitney test. Magnifications: × 20

In a PDX iCCA model that we have generated [11], *THY1*/CD90 was significantly downregulated at the transcriptional and protein level (*p* < 0.01 and *p* < 0.001, respectively), following treatment with Crenigacestat (Fig. 2A-B). These data suggest that *THY1*/CD90 expression in iCCA is regulated by the NOTCH1 inhibitor Crenigacestat.

**Fig. 3:**
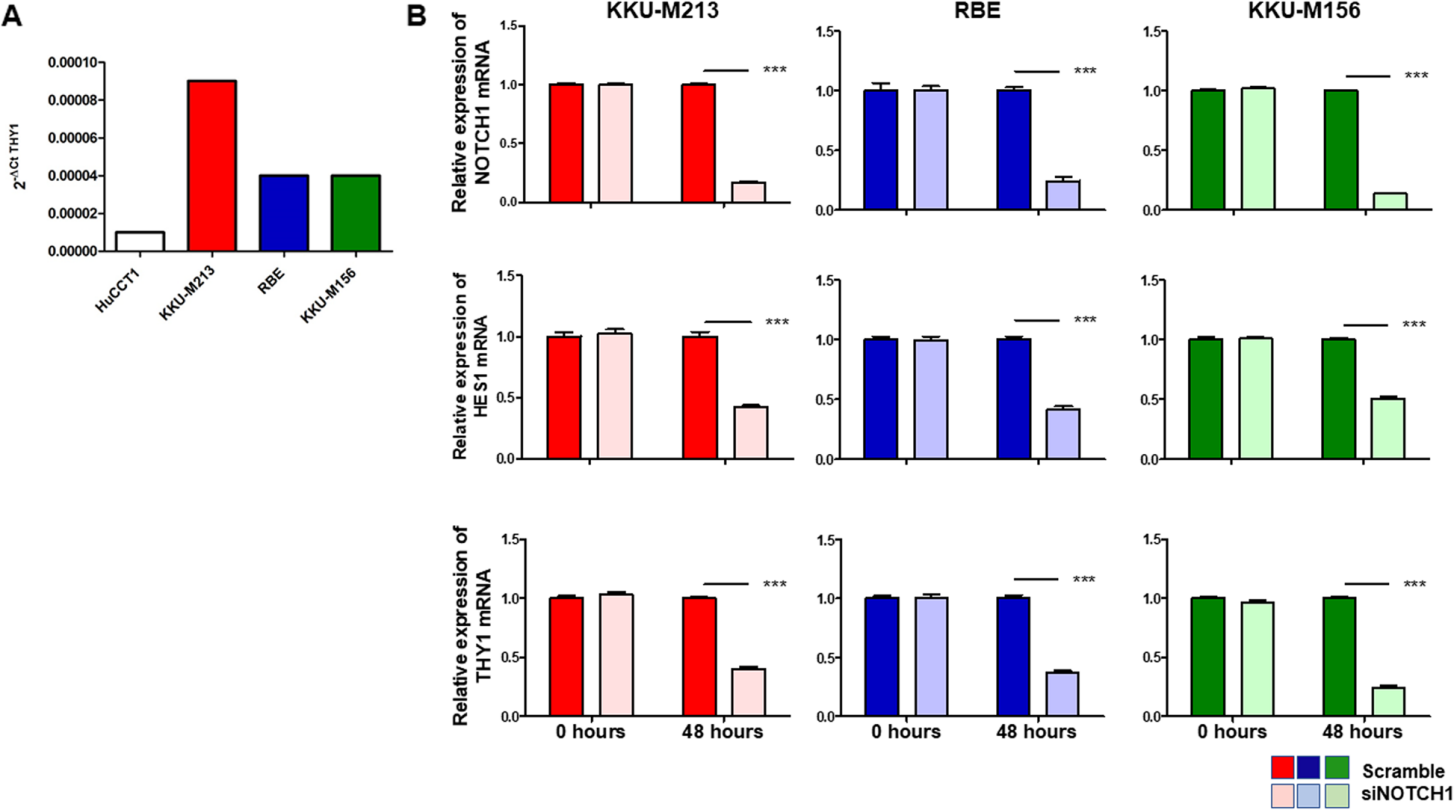
The Notch1 pathway regulates THY 1 expression in human iCCA cell lines. **A** KKU-M213, RBE, and KKU-M156 cells express constitutively high levels of THY mRNA, whereas HuCCT1 cells express very low levels. **B** KKU-M156, KKU-M213, and RBE Notch1 silenced cells expressed significantly lower THY mRNA levels than controls. ***p < 0.001 calculated with Student's t-test

To confirm our previous observation, we firstly checked the presence of CD90 in different iCCA cell lines and detected a strong expression of *THY1* mRNA in KKU-M213, RBE, and KKU-M156 versus a very weak expression in HuCCT1 (Fig. 3A). Then, to test the hypothesis that Crenigacestat inhibits *THY1*/CD90, we silenced NOTCH1 in the three cell lines highly expressing *THY1*. In all these cells, after knocking down *NOTCH1*, the canonical NOTCH target *HES1* was also strongly decreased (*p* < 0.001), and as hypothesized, *THY1* mRNA was also significantly (*p* < 0.001) downregulated (Fig. 3B). These results further support the role of NOTCH1 in modulating *THY1* expression.

**Fig. 4:**
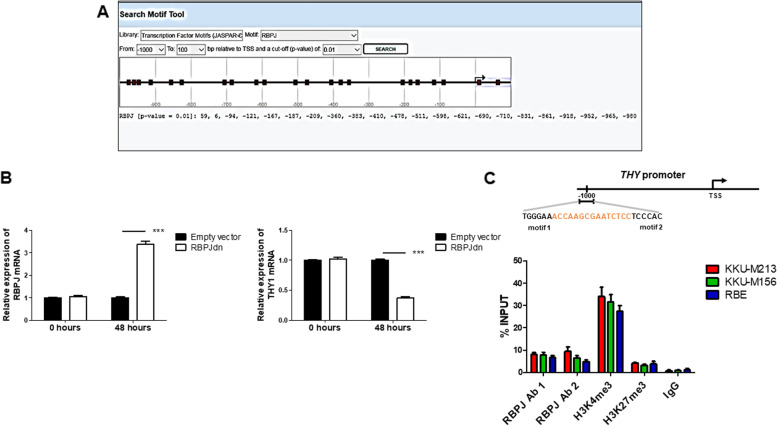
RBPJ specifically binds the THY1 gene promoter. **A** In-silico prediction of RPBJ binding sites on the THY1 gene promoter using the EPD new software, setting a P-value cut-off of 0.01. Binding sites are represented in red squares. **B** The Notch pathway was inhibited by transient overexpression of a dominant-negative form of the Notch transcriptional co-activator RBPJ (dnRBPJ). After transfection, THY1 gene expression levels were significantly reduced (***p < 0.001), showing that THY1 expression is downstream of the Notch1 pathway. **C** Chromatin Immunoprecipitation (ChIP) in KKUM-213, KKUM-156, and RBE cells. Chromatin was pulled down with anti-RBPJ (#1 and #2), -H3K4me3, and -H3K27me3 antibodies. Anti-IgGs were used as a negative control

Furthermore, in silico approaches revealed that RBPJ, an essential transcriptional regulator of the canonical NOTCH signaling, displays putative binding sites at the *THY1* gene promoter (Fig. 4A). To verify and validate this prediction, we inhibited the NOTCH pathway in vitro by transient overexpression of the dominant-negative form of *RBPJ* (*dnRBPJ*) [15, 17] in the representative RBE cell line. As expected, significant overexpression of *RBPJ* and a consistent reduction of *THY1* mRNA levels by qRT-PCR occurred (*p* < 0.001; Fig. 4B).

To further investigate the eventual binding of RBPJ at the *THY1* promoter, the in-silico analysis of the 5' UTR region of the *THY* locus was additionally analyzed. We found that the sequence-paired site (SPS) contains two *RBPJ* binding sites, at genomic positions 119,424,535 – 119,424,508 (Fig. 4C). This region is marked by activating epigenetic modifications typical of open chromatin, which is accessible to transcription machinery, as we identified an enrichment of the active marker H3K4me3 versus low amounts of the repressive marker H3K27me3. Of note, we discovered by chromatin immunoprecipitation that RBPJ binds to the detected SPS in iCCA cells, confirming our hypothesis that *THY1* is a downstream target gene of the NOTCH1 pathway.

### Crenigacestat inhibits the expression of THY1/CD90 in iCCA cells

**Fig. 5:**
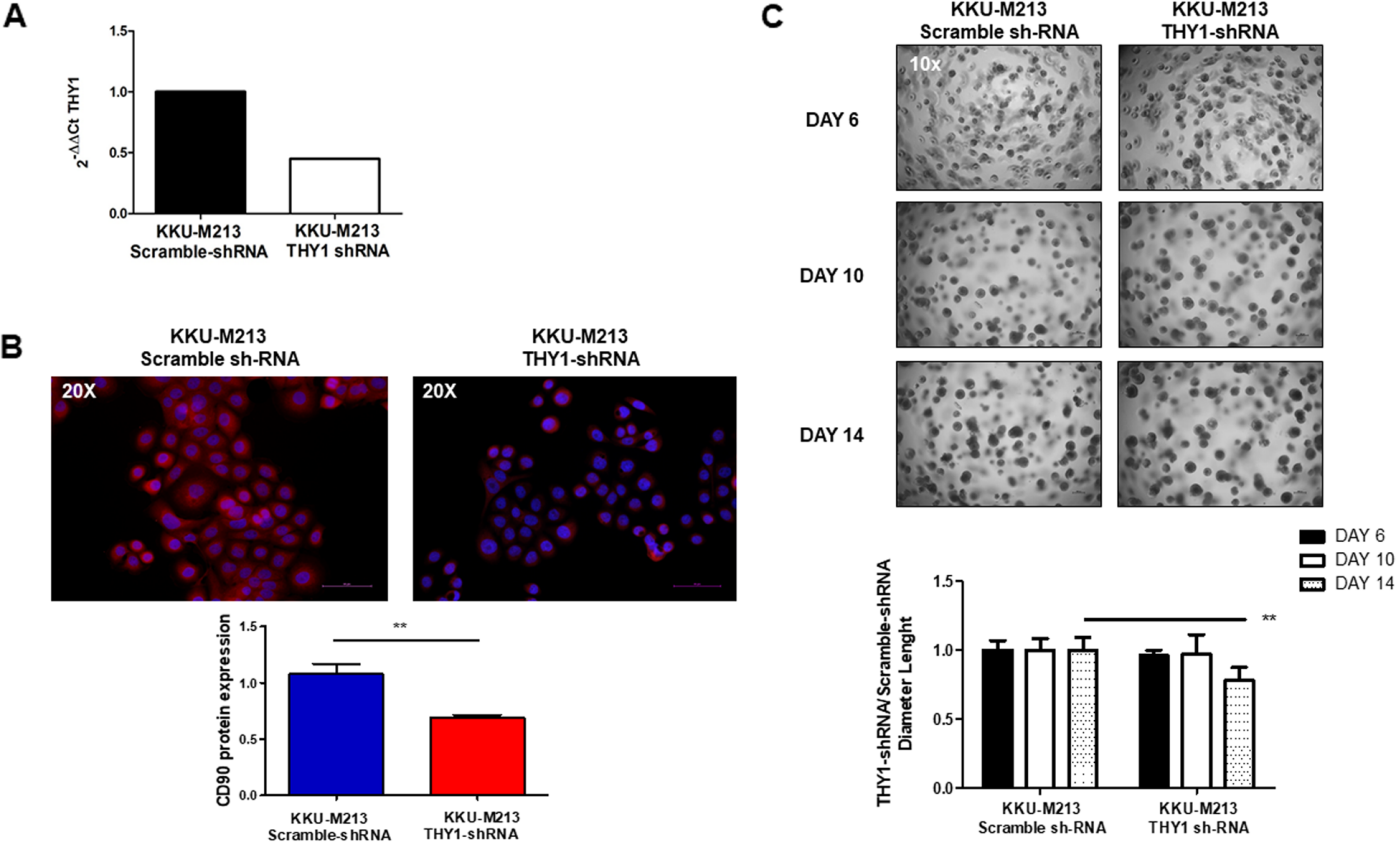
Functional study of KKU-M213 in vitro. **A-B** THY mRNA and CD90 protein levels were downregulated by 60% and 40% respectively, in silenced cells, as compared to sh-RNA cells.. **C** Representative spheroids images at 6–10-14 days of KKU-M213_scramble shRNA compared to KKU-M213_THY1/CD90-shRNA cells. Downregulation of THY1 expression significantly reduces spheroids of KKU-M213 cells. Spheroids diameter (μm) was measured and values reported in the graph below represent the mean ± SEM of three independent experiments relative to the control. **p < 0.01 calculated with Student’s t-test. (Magnification: 10 x)

To verify that Crenigacestat inhibits the NOTCH/*HES1*/CD90 axis in vitro, we used two different iCCA cell models engineered for loss or gain of CD90 expression. In the first case, we used KKU-M213 cells expressing constitutively high levels of *THY1*. At different Multiplicity of Infection (MOI) levels, we introduced lentiviral particles with scramble-shRNA or CD90-shRNA expression vector. *THY1*/CD90 was significantly (*p* < 0.001) inhibited at transcriptional and protein levels, as shown in Fig. 5A-B, in silenced transfected cells compared to scrambled transfected cells at MOI 100. In the functional assay, after 14 days, KKU-M213 scramble sh-RNA cells form spheres with a larger diameter than KKU-M213 THY1/CD90-shRNA, indicating that CD90 positive cells play a tumorigenic role, Fig. 5C.

**Fig. 6:**
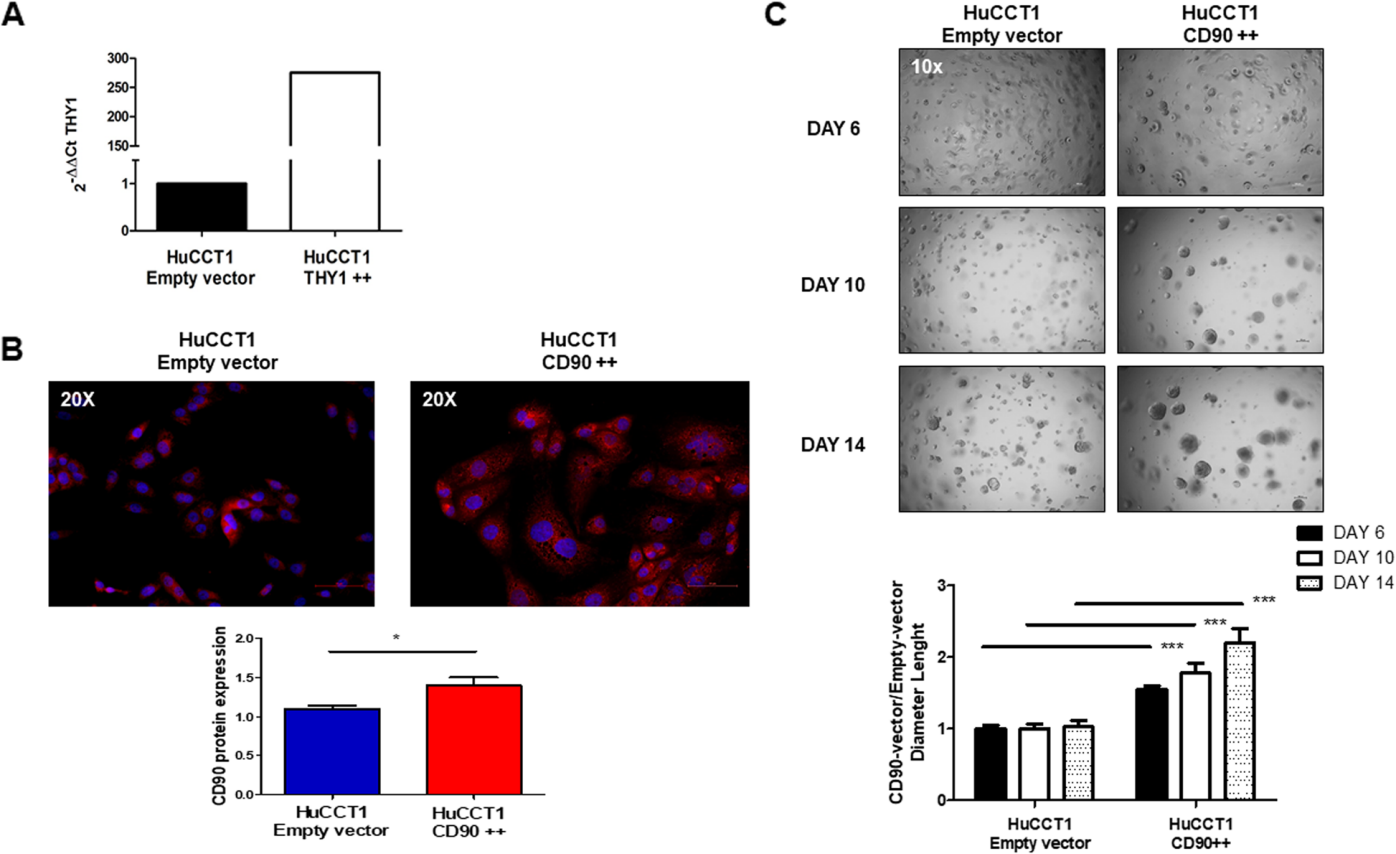
Functional study of HuCCT1 in vitro. **A-B** A 275-fold increase of mRNA expression and a threefold increase in protein levels are detected in HuCCT1 THY1/CD90 +  + compared to scrambled control. **C** Representative sphere-forming capacity images of HuCCT1_CD90 +  + compared to HuCCT1_empty vector at 6–10-14 days. Increased THY1 expression induces a greater number of tumor colonies and with a larger diameter, of HuCCT1 THY1/CD90 +  + cells. The diameter (μm) of the sphere was measured per microscopic field (Magnification: 10 x), and the values reported in the graph below represent the mean ± SEM of three independent experiments relative to the control. **p < 0.01; ***p < 0.001 calculated with Student’s t-test

In the second case, we used HuCCT1 cells previously shown to express constitutively low levels of CD90. As described above, we tested different MOI gain of *THY1*/CD90 expression, validated by qRT-PCR and immunofluorescence staining. The mRNA and protein expression levels of *THY1*/CD90 were significantly increased after the transduction approach in HuCCT1_ *THY1*/CD90 +  + at MOI 40 compared to those transduced with empty vector (*p* < 0.05; Fig. 6A-B). In line with previously described results, HuCCT1 THY1 +  + cells formed spheres, after 14 days, significantly faster and with a larger diameter, than HuCCT1 empty vector, supporting the conclusion that CD90 plays an essential role in maintaining iCCA tumor stemness, Fig. 6C.

**Fig. 7:**
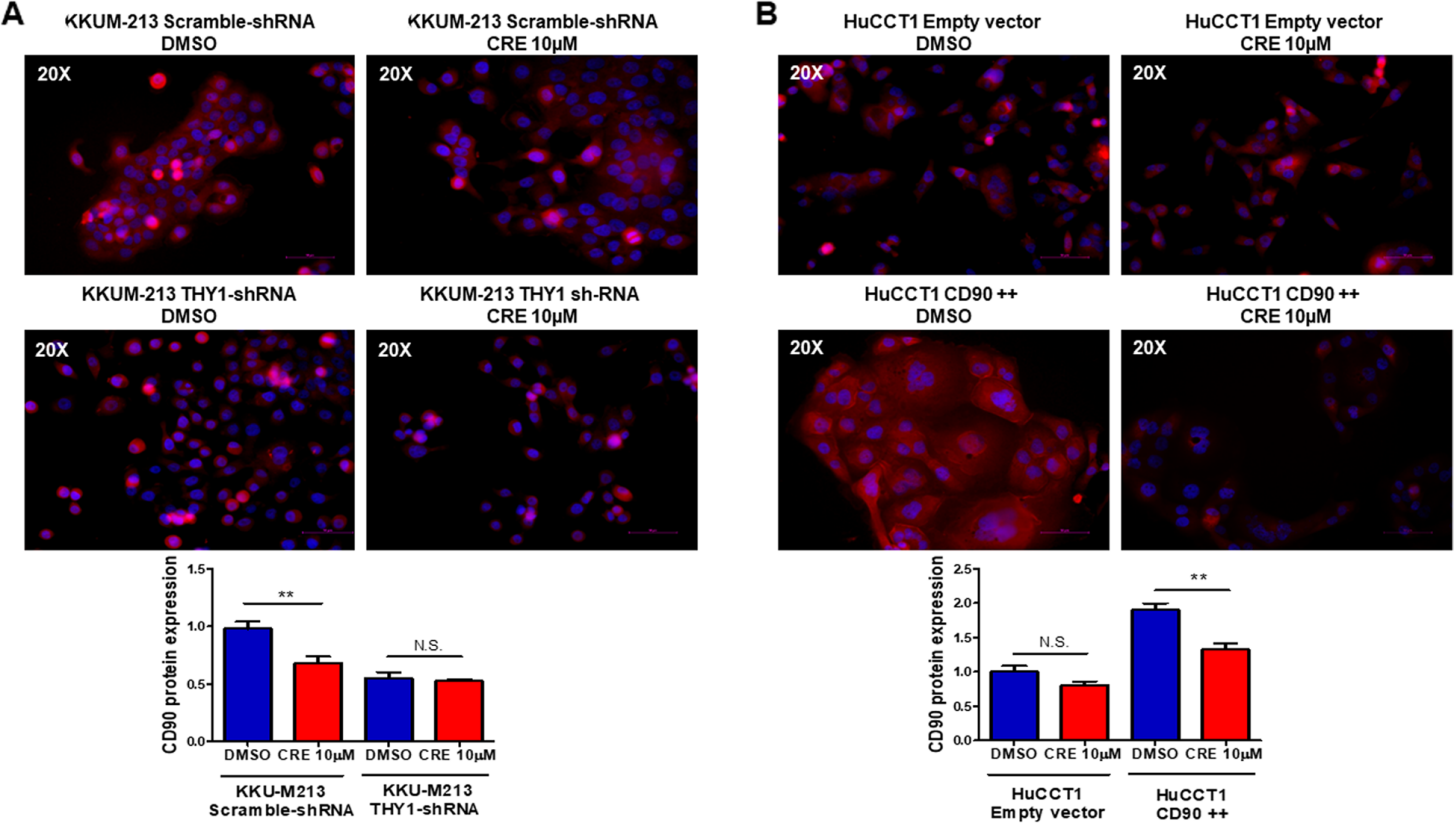
Crenigacestat decreases THY1 levels in CD90-positive cells. **A** Crenigacestat treatment reduces CD90 protein expression in KKU-M213_THY1-scramble sh-RNA but not in KKU-M213_THY1/CD90-shRNA cells. **B** Crenigacestat treatment significantly reduces CD90 protein expression in HuCCT1_CD90 +  + but not in the HuCCT1_empty vector cells. Magnification: × 20, N.S. No significant difference; **p < 0.01; calculated with Student’s t-test

Next, 10 µM Crenigacestat significantly (*p* < 0.01) inhibited CD90 levels in KKU-M213 scramble-shRNA but not in KKU-M213 *THY1*/CD90-shRNA cells (Fig. 7A). Also in this case, Crenigacestat 10 µM significantly (*p* < 0.01) reduced CD90 expression in the HuCCT1_CD90 +  + cells, but not in the HuCCT1 empty vector (Fig. 7B). These results suggest that Crenigacestat inhibits the NOTCH/*HES1*/CD90 axis in iCCA in vitro models.

### Crenigacestat inhibits tumor progression in CD90-positive xenograft models

To further demonstrate the effectiveness of Crenigacestat on iCCA expressing CD90, we used two complementary xenograft models built using the engineered iCCA cells with loss or gain of CD90 expression. First, stable KKU-M213 scramble-shRNA and KKU-M213 *THY1*/CD90-shRNA cells were engrafted in CD-1 nude mice. Then, after preliminary experiments to determine the growth curve, mice were treated 13 days after engraftment, when the tumor masses were sure to have grown, with vehicle or Crenigacestat at 8 mg/Kg (twice a week) for four weeks, monitoring the tumor growth twice a week.

**Fig. 8:**
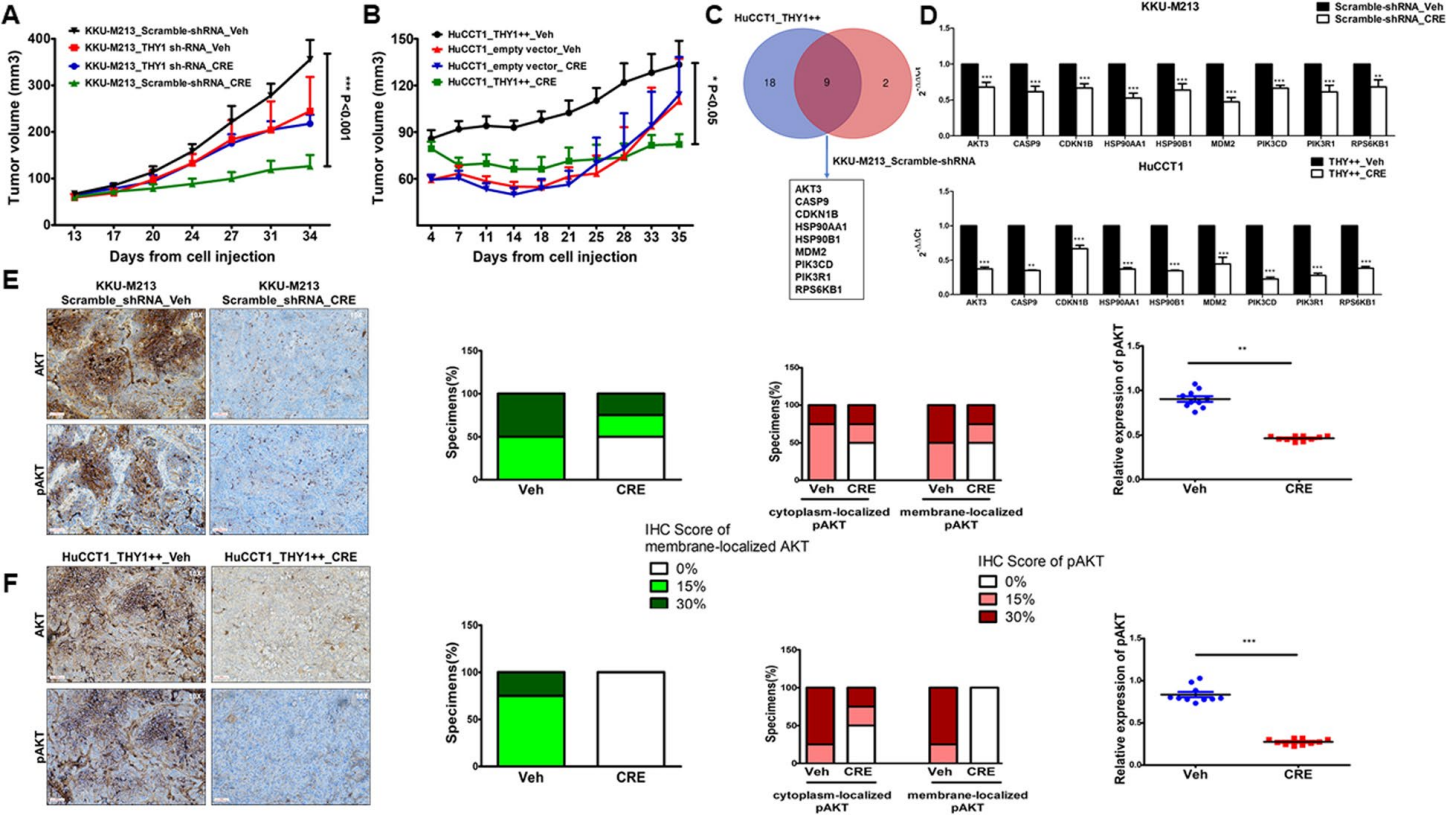
Crenigacestat inhibits the growth of CD90 positive xenograft tumors throughh the AKT signaling pathway **A** Four weeks' treatment with Crenigacestat significantly inhibited the growth of KKU-M213_scramble shRNA xenograft tumors compared with the same tumors treated with vehicle. On the contrary, no effect was observed in KKU-M213_THY1/CD90-shRNA following Crenigacestat treatment. **B** Growth of HuCCT1_THY1 +  + xenograft tumors was significantly reduced after 4 weeks of Crenigacestat treatment compared with the same tumors treated with vehicle. Crenigacestat did not display any effect on the Hucct1_empty vector cells. Each group consisted of 10 mice. *p < 0.05; ***p < 0.001 calculated with ANOVA test. **C** Venn diagram showing the number of common genes from the two comparisons. **D** PCR array analysis of 9 common genes in KKum213_scramble shRNA tumors (constitutively expressing high levels of CD90) treated with vehicle or Crenigacestat. PCR array analysis of 9 common genes in tumors originated from HuCCT1_CD90 +  + cells (transfected for overexpressing CD90) treated with vehicle or Crenigacestat. Histograms represent the mean ± SEM. *p < 0.05; **p < 0.01; ***p < 0.001. **E** Quantification and representative IHC staining of KKU-M213_scramble shRNA xenograft tumor treated with vehicle showing intense diffuse cytoplasmic positivity and complete circumferential membrane staining for 50% of AKT and 30% of pAKT. Weak cytoplasmic and membrane staining for 5% of AKT and pAKT in KKU-M213_scramble shRNA xenograft tumors treated with Crenigacestat. Scale bar 50 µM, Magnification 20X. Summary of semiquantitative analysis based on membrane localized-AKT and cytosolic or membrane pAKT expression in xenograft tumor masses with IHC score of positively staining cells: 0%; 15%; 30%. Scatter plot of pAKT expression level in tumors treated with vehicle or Crenigacestat obtained by measuring five microscopic fields randomly chosen for each section. Staining was calculated as mean ± SEM of the integrated density of pAKT normalized by the mean ± SEM of the integrated density of nuclei by Image J software on *n* = 10 KKU-M213_scramble shRNA xenograft tumors treated with vehicle and *n* = 10 KKU-M213_scramble shRNA xenograft tumors treated with Crenigacestat. **p < 0.01. **F** Quantification and representative IHC staining of HuCCT1_THY1 +  + xenograft tumors treated with vehicle showing intense circumferential complete positivity for 30% of AKT and pAKT. Absence of antigenic expression of AKT and pAKT in HuCCT1_THY1 +  + xenograft tumors treated with Crenigacestat. Scale bar 50 µM, Magnification 10X. Summary of semiquantitative analysis based on membrane localized-AKT and cytosolic or membrane pAKT expression in xenograft tumor masses with IHC score of positively staining cells: 0%; 15%; 30%. Scatter plot of pAKT expression level in tumors treated with vehicle or Crenigacestat obtained by measuring five microscopic fields randomly chosen for each section. Staining was calculated as mean ± SEM of the integrated density of pAKT normalized by the mean ± SEM of the integrated density of nuclei by Image J software on *n* = 10 HuCCT1_THY1 +  + xenograft tumors treated with vehicle and *n* = 10 HuCCT1_THY1 +  + xenograft tumors treated with Crenigacestat ***p < 0.001

KKU-M213 scramble shRNA tumors (constitutively expressing high levels of CD90) treated with vehicle grew faster than other experimental groups. However, Crenigacestat significantly (*p* < 0.001) inhibited the growth of KKU-M213 scramble shRNA tumors (expressing constitutively high levels of CD90) but not the growth of KKU-M213 *THY1*/CD90-shRNA. In controls, the vehicle did not affect the tumoral growth in either of the groups (Fig. 8A).

In a complementary mirrored experiment, HuCCT1 empty vector and HuCCT1 *THY1* +  + cells were engrafted in CD1 mice and treated with vehicle or Crenigacestat under the above described experimental conditions, after preliminary curve growth experiments. Consistently with the previous experiments, tumors originating from HuCCT1 *THY1* +  + cells (constitutively expressing low levels of CD90 and therefore transfected for CD90 overexpression) treated with vehicle showed the most aggressive phenotype. Moreover, Crenigacestat significantly (*p* < 0.01) reduced the growth of the HuCCT1 *THY1* +  + tumors, whereas it did not affect the growth of the HuCCT1 empty vector. In controls, the vehicle had no effect in either of the mice groups (Fig. 8B).

In conclusion, the combination of both experimental models demonstrates that iCCA tumors expressing CD90 display a more aggressive phenotype, and Crenigacestat inhibits the growth of tumors expressing high levels of CD90.

### CD90 affects iCCA progression by regulating the AKT signaling

To identify the downstream targets implicated in inhibiting tumor progression of xenograft models CD90 positive after Crenigacestat treatment, we used a commercially PCR array with 40 genes involved in the AKT signaling, frequently coactivated with NOTCH signaling in human iCCA [18–20].

In the KKU-M213_scramble shRNA tumors (constitutively expressing high levels of CD90) treated with vehicle or Crenigacestat, we found 11 genes significantly downregulated (≤ -1.5-fold change) in treated mice compared to vehicle (Supplementary Table 1). Complementarily, in tumors originating from HuCCT1_CD90 +  + cells (transfected to overexpress CD90), treated with vehicle or Crenigacestat, we found 27 genes significantly downregulated (≤ -1.5-fold change) in treated mice compared to vehicle (Supplementary Table 2). Then, we focused our attention on genes commonly modulated in both models. The Venn diagram revealed 9 common genes in the two models (Fig. 8C). Crenigacestat treatment reduced the expression of these genes when compared with their corresponding vehicle group (Fig. 8D).

As expected, Ingenuity pathway analysis (IPA) on these genes revealed that the PI3K/AKT signaling pathway was at the top of the significant pathways (Supplementary Fig. 1A). Moreover, the networks generated from the 9 genes were 2 and (Supplementary Fig. 1B-C), in both networks, downregulated genes were strongly interconnected and involved in cell proliferation and cancer.

According to bioinformatics and gene expression analyses, we performed immunohistochemistry to detect AKT and pAKT expression in CD90 positive xenograft tumors treated with vehicle or Crenigacestat. The levels of AKT and pAKT were strongly (66,99%, *p* < 0.001) and moderately (48,55%, *p* < 0.001) reduced by Crenigacestat treatment, respectively, in HuCCT1_THY1 +  + (Fig. 8E) and KKU-M213_scramble shRNA (Fig. 8F) xenograft tumors compared to vehicle.

Overall, we believe that the improvement response to Crenigacestat treatment in CD90 positive cells could be at least partly due to the inhibition of AKT signaling.

### Prognostic role of the NOTCH1/HES1/CD90 axis in iCCA patients

**Fig. 9:**
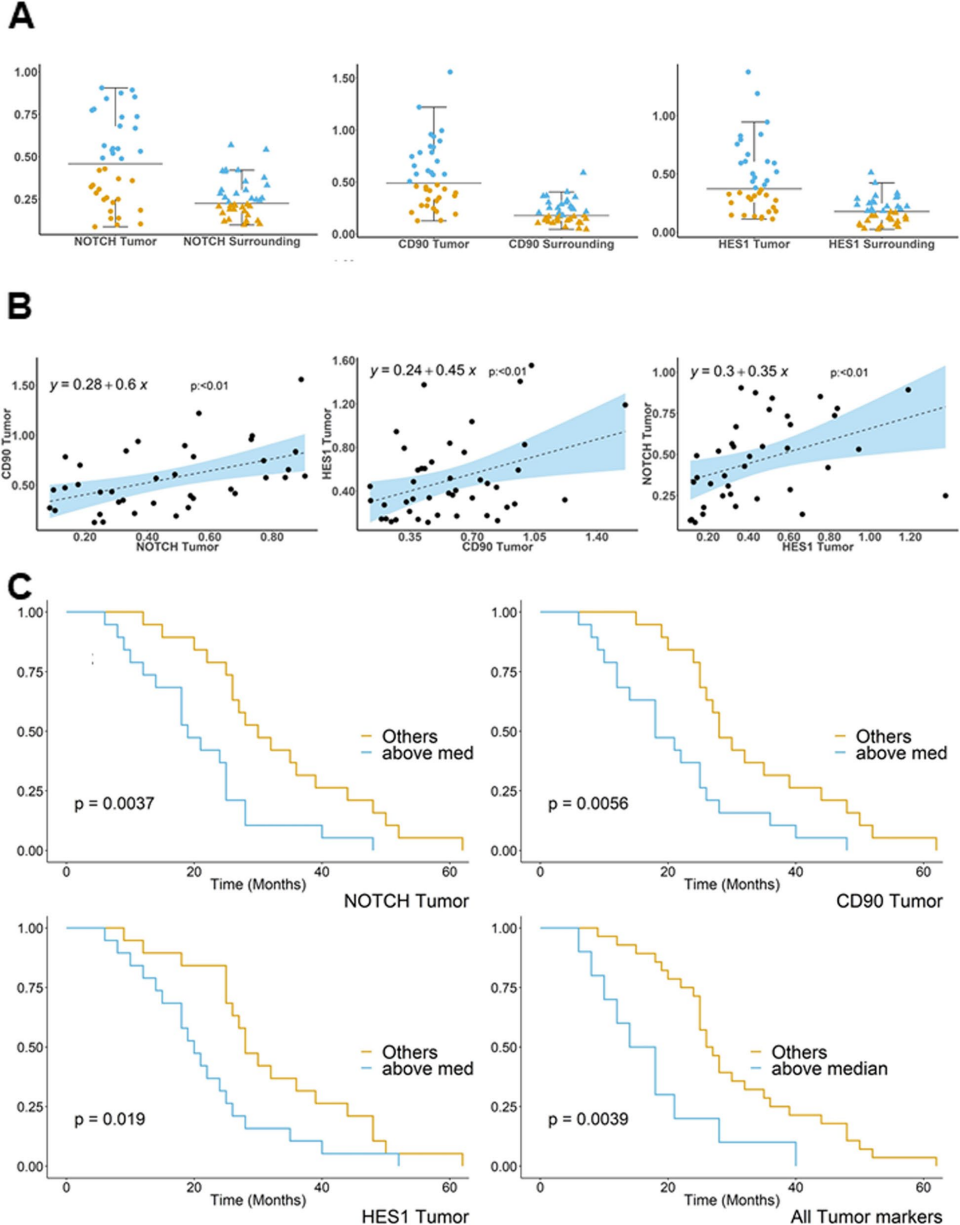
Jitter Boxplots and scatterplots of NOTCH1, CD90, and HES1. **A**. Boxplots of different quantities of biomarkers (y-axis) by tumor and tumor surrounding localization (x-axis), showing a jitter distribution (dot for tumor and triangle for surrounding tissue), median and interquartile range differences. **B** Scatter plots of correlation between the different tumor markers (NOTCH1, CD90, and HES1) showing the trend line (dotted line) and confidence intervals (blue area) and scatter interpolated distribution of the quantities (dots). **C** Kaplan Meier survival plots showing survival proportion of subjects above the median for each biomarker (orange lines) vs. subjects below the median (blue line) on the y-axis and survival time (months) on the x-axis

The analysis of RNA sequencing expression data on 31 iCCA patients' tissues and matched surrounding normal liver tissues from the GEO database (GSE107943) [21] showed that *THY1* mRNA levels were significantly higher in tumors compared to adjacent non-neoplastic tissues (*p* = 1.28*10^–7^) (Supplementary Fig. 2). In our population of 44 iCCA patients, for which clinicopathological data were available, with a mean age of 66.36 years, the gender distribution was 61% (*n* = 27) males and 39% (*n* = 17) females; 41% (*n* = 18) of the patients had evidence of cirrhosis, and in just 18% (*n* = 8), tumor size was greater than 5 cm. Moreover, 50% (*n* = 19) and 18% (*n* = 8) of patients had lymph nodes and lung metastasis, respectively, as reported in Supplementary Table 3. To test differences in the concentration of each biomarker between the tumor tissue and the surrounding area, we plotted the median distribution differences (and their interquartile ranges) of *NOTCH1, HES1*, and *THY1* in those areas in the whole population. The plots showed a statistically significant (*p* < 0.05) higher expression of each biomarker in the tumoral than in the peritumoral tissues (Fig. 9A). In addition, to assess the association between each biomarker present in the tumor tissue, we ran Pearson correlation analysis (Fig. 9B), demonstrating a positive, statistically significant association between any combination of them (*p* < 0.01) in the whole sample collection.

Finally, we investigated the prognostic power of *NOTCH1*, *HES1,* and *THY1*, alone or in combination, on iCCA patients' survival in months. As reported in Fig. 9C, Kaplan Meier survival analysis demonstrated that patients with *NOTCH1/HES1/THY1* expressions above the median values had worse survival than patients with values below the median (log-rank *p* < 0.05). In addition, survival was significantly shorter in months when considering the presence of each biomarker above the median (mean 17.5 ± 10.28, *p* < 0.05) than in patients without this condition (mean 30.36 ± 12.91). In conclusion, the presence of the *NOTCH1/HES1/THY1* axis defines a subset of iCCA patients (27%) with a more aggressive tumoral phenotype and a worse prognosis.

## Discussion

The minimal effectiveness of currently used chemotherapy and fast growth make iCCA a very deadly cancer. The poor knowledge of the molecular mechanisms regulating tumor-host interactions hampers the development of target-oriented therapies. In this study, for the first time, we show that CD90 is directly modulated by NOTCH and that a NOTCH1 γ-secretase inhibitor effectively reduces the progression of iCCA expressing high levels of CD90. We based this conclusion on the following data: 1) silencing of *NOTCH1* leads to a substantial reduction of *THY1* in iCCA cells constitutively expressing high levels of CD90; 2) inhibiting the NOTCH pathway by transient overexpression of the dominant-negative form of the RBPJ transcription factor, triggers a significant reduction of *THY1* mRNA levels; 3) in a xenograft model, Crenigacestat inhibits tumoral growth of KKU-M213 iCCA cells constitutively expressing high levels of CD90 but is not effective on the same cells after CD90 knockdown; 4) in a second xenograft model, Crenigacestat inhibits tumor growth of CD90-transfected HuCCT1 iCCA cells, but is not effective on the control cells constitutively expressing low levels of CD90.

Our results demonstrate that *THY1* is regulated by the NOTCH pathway and not vice versa, as described in K. Wu et al. [22] described in gastric cancer. In addition, as reported in several studies, DNA-binding protein RBPJ is a downstream transcription factor that occupies consensus DNA-binding sites while exchanging repressors for activators in response to NICD [23–26]. In our work, inhibition of the NOTCH pathway by dnRBPJ overexpression decreased *THY1* mRNA levels. Specifically, RBPJ is recruited to the *THY1* gene promoter via two *RBPJ* binding sites separated by spacer sequences of 15–17 base pairs (bp), enabling cooperative binding of NOTCH transcription complex dimers. Thus, the present data indicate that *THY1* expression is downstream of the NOTCH1 pathway. Similarly, K. Wu and colleagues reported in their work that upregulation of miR-140-5p inhibits the NOTCH signaling pathway and consequently reduces *THY1* expression in gastric cancer [22]. *THY1*/CD90 has been suggested as a microenvironment sensor in different cancers, including lung and glioblastoma [27, 28]. In HCC, CD45-CD90 + cells but not CD90 − cells isolated from blood and human tumor tissues generate tumor nodules in Beige/SCID mice. The authors subsequently demonstrated that the additional stem cell marker CD44 on the CD90^+^ cells contributed to greater aggression and metastasis of HCC subpopulations of CSCs [29]. Moreover, we demonstrated that the response to a NOTCH1 γ-secretase inhibitor treatment in CD90 positive cells could be mediated by the AKT signaling, specifically through AKT suppression. Indeed, NOTCH/AKT cross-regulation is crucial during iCCA development, achieved by hydrodynamic injection into the liver of an activated form of AKT in association with JAGGED1 or NOTCH1 [18, 19]. Herein, we demonstrate that Crenigacestat effectiveness in CD90 positive cells could be mediated by AKT signaling, in agreement with the study by Gao et al.[20], in which the authors showed that CD90 affects biological behavior and levels of energy metabolism of gastric cancer cells through activation of the PI3K/AKT/HIF-1α signaling pathway.

In iCCA patients, CD90 has been reported in human surgical samples and correlated with lymph nodes metastasis, indicating poor prognosis [10]. In a GEO database cohort, as well as in our collection of iCCA human tissue samples, *THY1* is more strongly expressed in tumoral compared to adjacent non-tumorous tissues. Herein, in a cohort of 44 patients, we demonstrate that *NOTCH1*, *HES1*, and *THY1* mRNA levels are positively correlated and are more pronouncedly expressed in tumoral than in peritumoral tissue. In this context, the Kaplan Meier survival analysis of human iCCA tumors indicated that patients with high *NOTCH1*, *HES1*, and *THY1* expression have the worst prognosis. Our findings are concordant with an increasing body of evidence strongly suggesting that the NOTCH signaling pathway is a prominent driver of CCA progression and poor prognosis [19, 30, 31]. However, the importance of *THY1* expression in human cancers is controversial. Indeed, except for human ovarian cancer and nasopharyngeal carcinoma, where CD90 seems to play a tumor suppressor role [32, 33], several works reported that CD90 overexpression in the tumor microenvironment enhances proliferation and metastasis, conferring the poorest outcome [10, 34–36]. Particularly, THY1 is highly expressed in cancer stem cells of gastric cancer, differentiated acute myeloid leukemia (AML) subtypes, lymph nodes metastasis in esophageal squamous carcinoma cells and also contributes to poor survival in HCC [8]. Here, we found an intriguing relationship between NOTCH signaling and *THY1*, closely associated with poor prognosis in patients with iCCA. According to the Luo et al. study, NOTCH1 signaling activated by JAGGED1 is positively related to CD90 + HCC CSCs with a rapid G1/S transition in the cell cycle phase [37]. Additionally, our results demonstrated that CD90 hallmarks a group of iCCA with a more aggressive phenotype responsive to Crenigacestat treatment. In this scenario, soluble THY1 has been detected in serum, cerebrospinal fluid, wound fluid, urine, and cerebrospinal fluid under normal and pathological conditions [38–41]. Our data suggest the measurement of THY1 in the bile fluid as a potential biomarker to select patients suitable for Crenigacestat treatment in future clinical trials. The identification of predictive biomarkers of response or survival such as *NOTCH1*, *HES1,* and *CD90* offers hope to patients with iCCA eligible for and likely to benefit from Crenigacestat therapy. Offering a chance to take a step toward a molecular classification of iCCA, it has been recently reported that approximately 8% of the patients carry fibroblast growth factor receptor-2 alterations and display a response rate higher than 35% to treatment with selective inhibitors [42]. Another 14% of the patients have isocitrate dehydrogenase gene alterations, and treatment with their inhibitors improved survival [43]. Finally, it has been reported that approximately 30–40% of the patients show a higher expression of VEGF, associated with the worst prognosis [44], while herein we report that 27% of the patients have higher levels of the NOTCH1/HES1/THY1 axis, correlated with shorter survival.

## Conclusions

Overall, our results highlight that patients with higher NOTCH1/HES1/CD90 expression have the worst prognosis and shorter survival. Nevertheless, in preclinical experimental models, this newly identified axis is successfully druggable with a NOTCH γ-secretase inhibitor, suggesting CD90 as a promising molecular target for treatment in a subset of patients with this disease.

To our knowledge, this is the first demonstrated evidence for a molecular stratification of iCCA predicting clinical outcomes. The successful use of the NOTCH1 γ-secretase inhibitor in preclinical models represents the scientific rationale for using this molecule in future clinical trials. In addition, this study takes the first step toward personalized medicine for patients currently lacking effective treatments.

## Supplementary Information

**Additional file 1.**

**Additional file 2.**

## Data Availability

All data generated or analysed during this study are included in this manuscript [and its supplementary information files].

## Abbreviations

CAFs: Cancer associated fibroblasts
CSC: Cancer stem cells
dnRBPJ: Dominant negative form of RBPJ
GSI: γ-Secretase inhibitor
HCC: Hepatocellular carcinoma
iCCA: Intrahepatic cholangiocarcinoma
MOI: Multiplicity of Infection
NCDI: NOTCH Intracellular Domain
PDX: Patient-Derived Xenograft
RBPJ: Recombination signal-Binding Protein for the immunoglobulin Kappa J region
siRNAs: Small interfering RNAs
THY1: Thymus cell antigen 1
