# Correction: CD90 is regulated by notch1 and hallmarks a more aggressive intrahepatic cholangiocarcinoma phenotype

**DOI:** 10.1186/s13046-023-02661-w

**Published:** 2023-04-18

**Authors:** Serena Mancarella, Grazia Serino, Isabella Gigante, Antonio Cigliano, Silvia Ribback, Paola Sanese, Valentina Grossi, Cristiano Simone, Raffaele Armentano, Matthias Evert, Diego F. Calvisi, Gianluigi Giannelli

**Affiliations:** 1 National Institute of Gastroenterology, IRCCS- Saverio de Bellis Research Hospital, Via Turi 27, 70013 Castellana Grotte, Italy; 2 Institute of Pathology, University of Regensburg, 93053 Regensburg, Germany; 3 Institute of Pathology, University of Greifswald, 17489 Greifswald, Germany

**Correction** **:** **J Exp Clin Cancer Res 41, 65 (2022)**

**https://doi.org/10.1186/s13046-022-02283-8**

Following publication of the original article [1], an error was identified in the corresponding author’s affiliation.

The updated affiliation is given below and the changes have been highlighted in **bold typeface**.

^1^National Institute of Gastroenterology, **IRCCS- Saverio de Bellis** Research Hospital, Via Turi 27, 70013 Castellana Grotte, Italy.

The correction do not affect the overall Conclusion of the article.
